# Effects of Methylenediphenyl 4,4’-Diisocyanate and Maleic Anhydride as Coupling Agents on the Properties of Polylactic Acid/Polybutylene Succinate/Wood Flour Biocomposites by Reactive Extrusion

**DOI:** 10.3390/ma13071660

**Published:** 2020-04-03

**Authors:** Young-Rok Seo, Sang-U Bae, Jaegyoung Gwon, Qinglin Wu, Birm-June Kim

**Affiliations:** 1 Department of Forest Products and Biotechnology, Kookmin University, Seoul 02707, Korea; kurirok2@kookmin.ac.kr (Y.-R.S.); bsangu6571@kookmin.ac.kr (S.-U.B.); 2 Wood Chemistry Division, National Institute of Forest Science, Seoul 02455, Korea; gwonjg@korea.kr; 3 School of Renewable Natural Resources, Louisiana State University AgCenter, Baton Rouge, LA 70803, USA; QWu@agcenter.lsu.edu

**Keywords:** polylactic acid, polybutylene succinate, wood flour, coupling agent, isocyanate, maleic anhydride, properties

## Abstract

Polylactic acid (PLA)/polybutylene succinate (PBS)/wood flour (WF) biocomposites were fabricated by in situ reactive extrusion with coupling agents. Methylenediphenyl 4,4’-diisocyanate (MDI) and maleic anhydride (MA) were used as coupling agents. To evaluate the effects of MDI and MA, various properties (i.e., interfacial adhesion, mechanical, thermal, and viscoelastic properties) were investigated. PLA/PBS/WF biocomposites without coupling agents revealed poor interfacial adhesion leading to deteriorated properties. However, the incorporation of MDI and/or MA into biocomposites showed high performances by increasing interfacial adhesion. For instance, the incorporation of MDI resulted in improved tensile, flexural, and impact strengths and an increase in tensile and flexural modulus was observed by the incorporation of MA. Specially, remarkably improved thermal stability was found in the PLA/PBS/WF biocomposites with 1 phr MDI and 1 phr MA. Also, the addition of MDI or MA into biocomposites increased the glass transition temperature and crystallinity, respectively. For viscoelastic property, the PLA/PBS/WF biocomposites with 1 phr MDI and 1 phr MA achieved significant enhancement in storage modulus compared to biocomposites without coupling agents. Therefore, the most balanced performances were evident in the PLA/PBS/WF biocomposites with the hybrid incorporation of small quantities of MDI and MA.

## 1. Introduction

Recently, environmental pollution caused by petrochemical-based polymers has attracted much attentions because it is an important issue in daily life. As a solution to this problem, there is a growing interest in biodegradable polymers due to the advantage of biodegradability after use [1,2,3].

Polylactic acid (PLA) is a common aliphatic polyester thermoplastic polymer derived from renewable resources such as corn starch, tapioca roots, sugar cane, and sugar beet [4]. PLA has been applied to various industries because of its biodegradability, ease of processing, good mechanical property, biocompatibility, and transparency [5]. However, its disadvantages of brittleness, low melt strength, reduced durability, and low thermal stability limit its application in advanced industries [5,6]. One way to improve the limited properties of PLA can be demonstrated by mixing it with other biodegradable polymers [7]. Polybutylene succinate (PBS) is an aliphatic polyester thermoplastic polymer having good processing stability, high flexibility, excellent impact strength, good elongation at break, and excellent biodegradability. The combination of PLA and PBS was expected to improve the limited properties of PLA, including its low impact strength, reduced durability, and low thermal qualities [8].

Biodegradable polymers are becoming increasingly important; however, as they are costlier than conventional petro-based polymers, to have cost competitiveness, there is a need for the study of biodegradable polymer-based composites filled with natural resources. Wood flour (WF) is a lignocellulosic resource derived from wood; it is globally abundant, inexpensive, very light, and biodegradable [9]. However, the surface of WF has a hydrophilic functional group that exhibits poor interfacial adhesion when blended with hydrophobic polymers (such as PLA and PBS). This leads to low dispersion, high viscosity, and reduced compatibility [10]. Poor interfacial adhesion between the polymer matrix and the filler results in reduced mechanical and thermal properties. Thus, simple mixing of PLA, PBS, and WF to produce composite materials is an ineffective methods.

Coupling agents (or interfacial modifiers) with reactive functional groups induce the production of grafts or copolymers in situ to improve the miscibility between polymer matrix and filler. Previous studies have examined the properties of methylenediphenyl 4,4’-diisocyanate (MDI) as a coupling agent to improve the interfacial compatibility between polymer matrix and natural fillers. As a coupling agent for composites, MDI reacts mainly with hydroxyl or amino groups to form stable chemical bonds [11,12]. Hence, MDI is incorporated into biocomposites to induce excellent interfacial adhesion between PLA, PBS, and WF with hydroxyl groups. Darie et al. reported that the incorporation of toluene-2,4’-diisocyanate improved the interfacial adhesions between PLA, polypropylene, and eucalyptus wood fiber, thereby demonstrating good mechanical properties [13]. Li et al. also found that the incorporation of MDI improved the miscibility between PLA and PBS, which resulted in increased elongation at break [14]. Additionally, Pan et al. demonstrated successful improvements of impact strength and thermal behavior for PLA and poly(butylene adipate-co-butylene terephthalate) blends with MDI [15]. Maleic anhydride (MA) is one of the most widely used reactive coupling agents due to its good chemical reactivity under free radical grafting conditions [16]. MA reacts with a polymer to form a maleated polymer (which improves its miscibility with natural fillers); this is used mainly in the reaction of polyolefin-based polymers, such as polyethylene and polypropylene [17]. Anhydride groups of the maleated polymer react with the hydroxyl groups of the natural filler to form cross-linked hydrogen or ester type bonds [18]. Although MA is used mainly in polyolefin-based polymers, many studies have applied it as a coupling agent for polyester-based polymers (such as PLA and PBS). Lv et al. reported that the addition of MA into PLA/WF composites improved tensile strength, flexural strength, and water resistance by enhancing interfacial adhesion [18]. In addition, Liminana et al. noted that PBS / almond shell flour composites prepared with MA showed the most balanced improvement by increasing mechanical, thermal and thermodynamic properties [19]. Although many studies have been carried out by incorporating natural fillers into PLA or PBS polymer matrix, very little work has been done on PLA/PBS/WF ternary blends with coupling agents.

In this study, PLA/PBS/WF biocomposites with coupling agents were fabricated by one-step reactive extrusion without additional processing, which involved an injection molding process after compounding by twin-screw extrusion. The main objective of this study is to evaluate the effects of MDI and MA as coupling agents on the interfacial adhesion, mechanical, thermal, and viscoelastic properties of PLA/PBS/WF biocomposites.

## 2. Materials and Methods

### 2.1. Materials

PLA (4043D), with a melting flow index of 6 g/10 min (210 °C/1.96 kg) and a density of 1.24 g/mL, was obtained from NatureWorks, LLC, Minnetonka, MN, USA. PBS (G4560M), with a melting flow index of 3 g/10 min (190 °C/2.16 kg) and a density of 1.24 g/mL, was obtained from S-EnPol Co., Ltd., Wonju, South Korea. WF (arbocel C100) was supplied by J. Rettenmaier & Söhne, Rosenberg, Germany. It consisted mainly of Norway spruce (*Picea abies*), with an average grain size of 70–150 μm. MDI (purity > 97%) was purchased from Tokyo Chemical Industry Co., Ltd., Tokyo, Japan. MA was provided by Duksan Chemical Co., Ansan-si, South Korea. Dicumyl peroxide (DCP) was obtained from Acros Organics BVBA, Geel, Belgium and was used to initiate the grafting reaction of polymer and MA. The addition ratio of DCP was maintained as 0.1 phr per 1 phr of MA.

### 2.2. Methods

#### 2.2.1. Melt Compounding and Specimen Preparation

**Figure 1:**
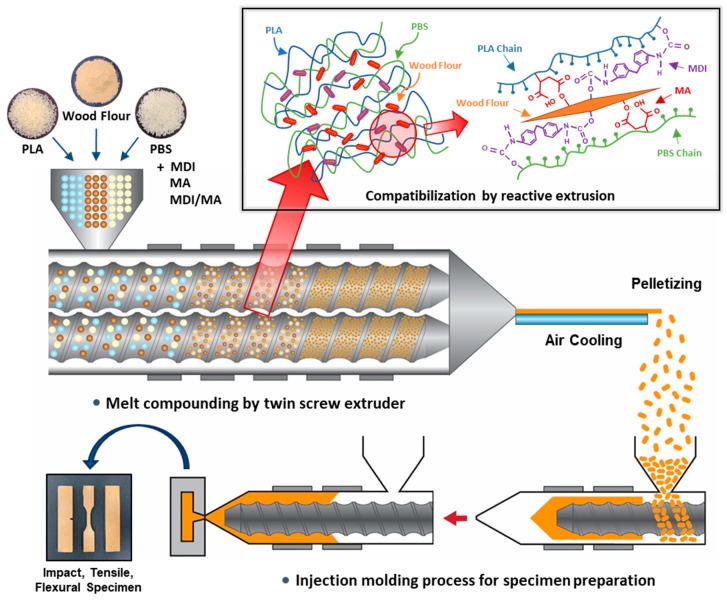
Schematic of overall biocomposites manufacturing process by reactive extrusion.

**Figure 2:**
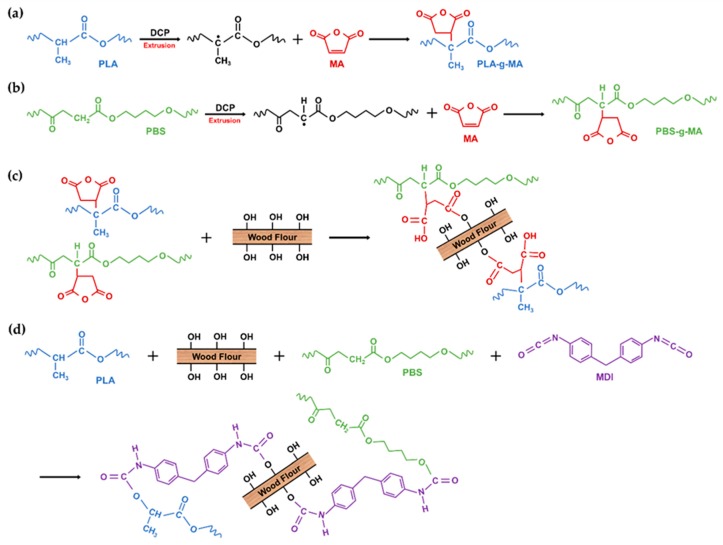
Schematic of overall chemical reaction mechanisms: (**a**) Interactions between PLA and MA; (**b**) Interactions between PBS and MA; (**c**) Interactions between PLA-g-MA, PBS-g-MA, and WF; (**d**) Interactions between PLA, PBS, WF, and MDI.

PLA and PBS were dried at 55 °C for 48 h and WF was dried at 80 °C for 48 h to remove the moisture from the raw materials before compounding. The dried materials were stored in a polyethylene bag. PLA, PBS, WF, and the MDI and MA coupling agents were melt-compounded using a BA-19 co-rotating twin-screw extruder (Bautek Co., Ltd., Uijeongbu, South Korea) with a length-to-diameter ratio of 40. The barrel temperature zones were set at 180, 185, 190, 185, 180, 170, and 120 °C, and the extruder rotation speed was 85–90 rpm. After air cooling, the extruded blends were pelletized and then the pellets were manufactured into the test specimens using a BOY 12M injection molding machine (Dr. Boy GmbH & Co. KG, Neustadt, Germany). A total of eight types of PLA/PBS/WF biocomposites were prepared in different formulation ratios of PLA, PBS, WF, MDI, MA and DCP. The formulation ratios for the PLA/PBS/WF biocomposites are shown in Table 1. The overall processing procedure and the compatibilization effect in the melt extrusion process are shown in the schematic of Figure 1. In addition, the expected chemical reaction mechanism in the compatibilization process is shown in the schematic of Figure 2.

#### 2.2.2. Characterization of Composites

An attenuated total reflectance Fourier transform infrared (FT-IR) spectroscope was used to analyze the chemical composition changes of PLA/PBS/WF biocomposites by coupling agents. The spectra were collected over the wavelength range of 4000–600 cm^−1^ (32 scans) using a MIR-FIR FT-IR spectrometer (Perkin-Elmer, Waltham, MA, USA).

The morphologies of the PLA/PBS/WF biocomposites were characterized by using a JSM-7401F (Jeol Ltd., Tokyo, Japan) field-emission scanning electron microscope (SEM). After Izod impact tests, fractured surfaces of the specimens were observed. The fractured surfaces were platinum-coated and then analyzed at an acceleration voltage of 10 kV.

#### 2.2.3. Mechanical Properties

Izod impact strength tests were performed using a DTI-602B digital impact tester (Daekyung Technology, Incheon, South Korea) according to ASTM D256. Tensile and flexural strength tests were conducted using a H50KS universal testing machine (Hounsfield, Surrey, UK) according to ASTM D638 and ASTM D790, respectively. The test crosshead speed was set at 10 mm/min. At least five replicates were tested for each sample to obtain an average value.

#### 2.2.4. Thermal Properties

Thermogravimetric analysis (TGA) was carried out using a TGA/DSC 1 thermogravimetric analyzer (Mettler Toledo, Columbus, OH, USA) to investigate the thermal decomposition temperature of PLA/PBS/WF biocomposites. All specimens were heated in a nitrogen gas atmosphere from 30 to 600 °C at a heating rate of 10 °C/min. The weight of the specimens was kept at about 5 mg of extruded pellets.

Differential scanning calorimeter (DSC) analysis was conducted using a DSC Q10 instrument (TA Instruments, New Castle, DE, USA) to study the thermal behavior of the PLA/PBS/WF biocomposites. All specimens were heated from 30 to 250 °C at a heating rate of 10 °C/min; this was maintained for 1 min before being reduced at a cooling rate of −10 °C/min in a nitrogen gas atmosphere. The weight of the samples was kept at about 10 mg of extruded pellets. The values for glass transition temperature (*T*_g_), melting temperature (*T*_m_), and melting enthalpy (Δ*H*_m_) were determined from the heating scan. The crystallinity (*X_c_*) of PLA/PBS/WF biocomposites were calculated using the following Equation (1):

(1) $$\chi c=\frac{\Delta H_{m}}{\Delta {H_{m}}^{0}}\times 100\;\left(\%\right)$$

where Δ*H*_m_ is the melting enthalpy of the composite and Δ*H*_m_^0^ is the theoretical enthalpy of the PLA and PBS (PLA: 93.1 J/g, PBS: 110.3 J/g) [20].

#### 2.2.5. Viscoelastic Properties

Dynamic mechanical analysis (DMA) was performed using a TA Q800 dynamic mechanical analyzer (TA Instruments, New Castle, DE, USA) to investigate the viscoelastic properties of the PLA/PBS/WF biocomposites. A rectangular specimen was 35 × 12 × 3 mm in size. All specimens were measured using a dual cantilever method at a frequency of 1 Hz and a strain rate of 0.1%. All specimens were heated from −60 to 110 °C at a heating rate of 5 °C/min.

## 3. Results and Discussion

### 3.1. Characterization of Composites

**Figure 3:**
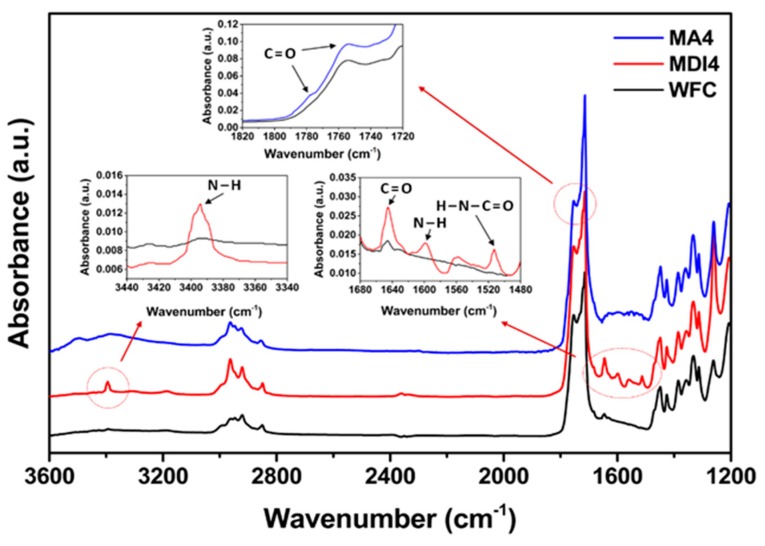
FT-IR spectra of various PLA/PBS/WF biocomposites with/without MDI or MA.

FT-IR spectra of PLA/PBS/WF biocomposites without coupling agents and with MDI or MA are shown in Figure 3. The presence of MDI in the biocomposites caused the appearance of a new absorption peak at 3394 cm^−1^ (N–H stretching) compared with the absence of MDI [15]. On the other hand, the stretching corresponding to the isocyanate group was not found at 2270 cm^−1^, indicating that the initial isocyanate groups completely reacted with PLA, PBS, and WF [15]. In addition, the peak at 1645 cm^−1^ represents the stretching of C=O in urethane groups (H-N-C=O stretching) and ester groups, and the peak at 1595 cm^−1^ is assigned the N-H stretching of urethane groups [21]. These peaks seem to be due to the reaction between isocyanate groups constituting MDI and hydroxyl groups constituting PLA, PBS, and WF, resulting in a chain extension effect to form urethane linkages. The presence of MA in the PLA/PBS/WF biocomposites revealed an increased stretching of the absorption peaks at 1750 and 1780 cm^−1^ (C=O stretching). The peak at 1750 cm^−1^ shows an increased absorption peak resulted from the introduction of ester groups by grafted MA [22]. This suggests that the carbonyl group comprising MA was appropriately grafted onto the polymer matrix, which indicates increased stretching. Meanwhile, the peak at 1780 cm^−1^ is due to free MA, and it is attributed to the unreacted free MA remaining in MA4 with the highest content of MA. Hence, it can be expected that the presence of excess MA may show rather deteriorated properties. The FT-IR spectra indicate that certain compatible structures could be achieved between PLA, PBS and WF through appropriate chemical reactions under melt-grafting conditions by reactive extrusion based on MDI and MA.

**Figure 4:**
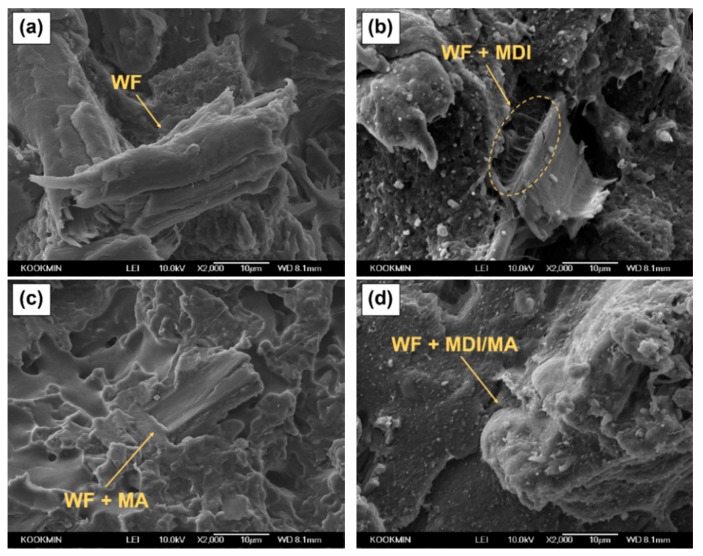
SEM micrographs of the impact-fractured surfaces (cross section of the specimen) of various PLA/PBS/WF biocomposites: (**a**) WFC, (**b**) MDI4, (**c**) MA4, and (**d**) MDI/MA4.

SEM images of various PLA/PBS/WF biocomposites are presented in Figure 4. As observed in Figure 4a, PLA/PBS/WF biocomposite without coupling agents shows that WF particle is isolated from polymer matrix. This is probably due to the poor interfacial adhesion between polymer matrix and WF, resulting in easy debonding during fracture. Figure 4b shows a SEM image of PLA/PBS/WF biocomposite with MDI. In this figure, interfaces between WF and polymer matrix are connected together by urethane linkages resulted from the reaction of isocyanate group constituting MDI [23]. In Figure 4c, PLA/PBS/WF biocomposites with MA shows smoother fracture surface and it seems that the unsaturated carbon–carbon bonds in MA chemical structure are likely to graft onto the surface of PLA and PBS, while the anhydride groups are likely to react with the hydroxyl groups of WF, resulting in improved interfacial adhesion [18]. Figure 4d is an SEM image of PLA/PBS/WF biocomposites with MDI and MA. It is similar to Figure 4b,c in that the interfacial adhesion between polymer matrix and WF was improved. Therefore, the MDI and MA played a significant role in improving the compatibility between PLA, PBS, and WF.

### 3.2. Mechanical Properties

**Figure 5:**
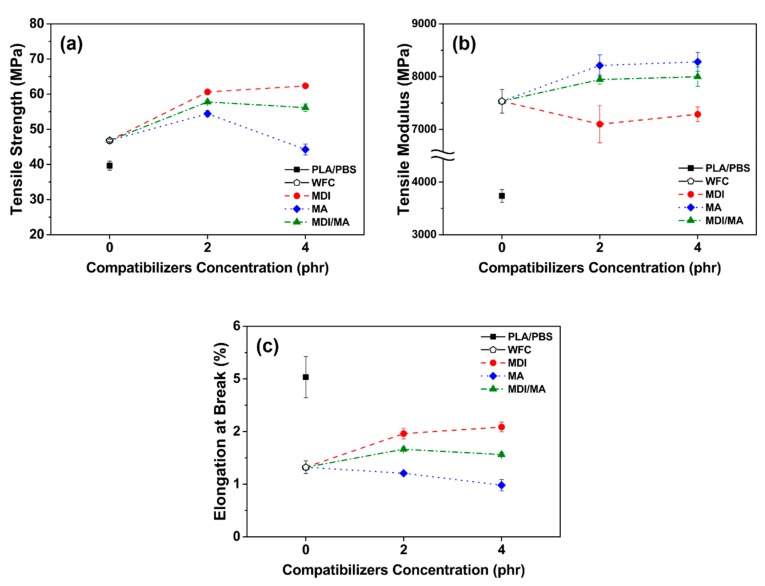
Tensile properties of various PLA/PBS/WF biocomposites: (**a**) Tensile strength, (**b**) tensile modulus, and (**c**) elongation at break.

**Figure 6:**
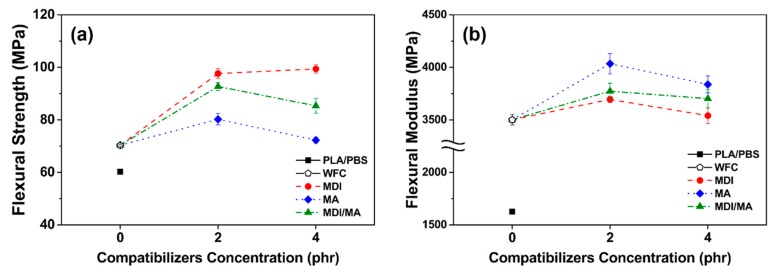
Flexural properties of various PLA/PBS/WF biocomposites: (a) flexural strength and (b) flexural modulus.

Tensile and flexural properties of various PLA/PBS/WF biocomposites are shown in Figure 5 and Figure 6. As observed in Figure 5a and Figure 6a, tensile and flexural strength improved with the incorporation of WF, most likely because of the increased orientation of WF in the longitudinal direction during the injection molding process and the interference of the appropriately dispersed WF. The incorporations of 2 phr MDI, MA, and MDI/MA into biocomposites resulted in tensile strength values of 60.20, 54.42, and 57.80 MPa, respectively. In flexural strengths, the addition of 2 phr coupling agents showed the strength values of 97.66, 80.21, and 92.71 MPa, respectively. As shown in Figure 5 and Figure 6, the increase in tensile and flexural strength values was the highest for the PLA/PBS/WF biocomposites with MDI and the lowest for the PLA/PBS/WF biocomposites with MA. This is because the interfacial adhesion was improved by the urethane linkages that were produced by the chemical reactions between isocyanate groups of MDI and hydroxyl groups of PLA, PBS, and WF [11,23]. Also, MA was grafted onto the PLA and PBS by reactive extrusion, which resulted in the chemical bonding between the anhydride group of MA and the hydroxyl groups of PLA, PBS, and WF. Additionally, the improved interfacial adhesion by the incorporation of coupling agents leads to increased tensile and flexural strength by protecting the molecular chains from slippage [18]. However, the incorporation of 4 phr MDI, MA, and MDI/MA resulted in tensile strength values of 62.35, 44.26, and 56.16 MPa, respectively, and flexural strength values of 99.40, 72.29, and 85.35 MPa, respectively. The incorporation of additional MDI similarly improved both tensile and flexural strength values; this observation was similar to the study by Lee et al. [24]. There is a maximum saturation concentration value for the surfactant action by the isocyanate of MDI. Thus, it can be said that the incorporation of additional MDI is ineffectual for PLA/PBS/WF biocomposites [11,25]. In the case of incorporation of additional MA, both the tensile and flexural strength values were remarkably decreased; this observation is similar to the research of Gunning et al. [26]. With an increased MA concentration, the excess MA monomer that remained at the interface between PLA, PBS, and WF made the molecular chains slide easily from each other and induced the decreases in the tensile and flexural strengths [18]. It is considered to exhibit rather deteriorated mechanical properties when excess MA is present, as mentioned in the FT-IR results. PLA/PBS/WF biocomposites with MDI/MA demonstrated balanced strength values due to the combined effect of both MDI and MA.

Tensile and flexural moduli values were improved significantly with the incorporation of WF as shown in Figure 5b and Figure 6b. The WF particles are considered to enhance the stiffness of PLA/PBS/WF biocomposites by limiting the movement of the polymer chain [27]. As observed in Figure 5b, the incorporation of 2 phr MDI, MA, and MDI/MA resulted in tensile moduli values of about 7098, 8212, and 7944 MPa, respectively. Compared to other PLA/PBS/WF biocomposites, relatively low tensile modulus value of PLA/PBS/WF biocomposite with MDI is probably associated with the increased ductility of the biocomposite. This can be noticed in relatively high elongation at break values of the PLA/PBS/WF biocomposites with MDI as shown in Figure 5c. On the other hand, the incorporation of MA led to enhanced tensile modulus due to the improved interfacial adhesion between PLA, PBS, and WF. At the 4 phr level of coupling agents, MDI, MA, and MDI/MA resulted in tensile moduli values of 7286, 8281, and 7999 MPa, respectively. These results represented a slight improvement, but demonstrated no significant differences. Similar to tensile strength, the tensile moduli of PLA/PBS/WF biocomposite with MDI/MA demonstrated a balanced feature between the moduli values of PLA/PBS/WF biocomposites with MDI or MA. Flexural moduli values of 3695, 4035, and 3773 MPa are represented in the PLA/PBS/WF biocomposites with 2 phr MDI, MA, and MDI/MA, respectively. These flexural moduli values were increased compared to that of PLA/PBS/WF biocomposite without coupling agents. It was most improved with the incorporation of MA and was slightly improved with the incorporation of MDI. From these results, it is considered that the cause was similar to that for the tensile moduli results. The PLA/PBS/WF biocomposites with 4 phr MDI, MA, and MDI/MA exhibited flexural moduli values of 3540, 3838, and 3702 MPa, respectively. The flexural moduli values were reduced by the incorporation of all coupling agents. This result means that excessive additions of coupling agents resulted in a negative effect in biocomposites.

**Figure 7:**
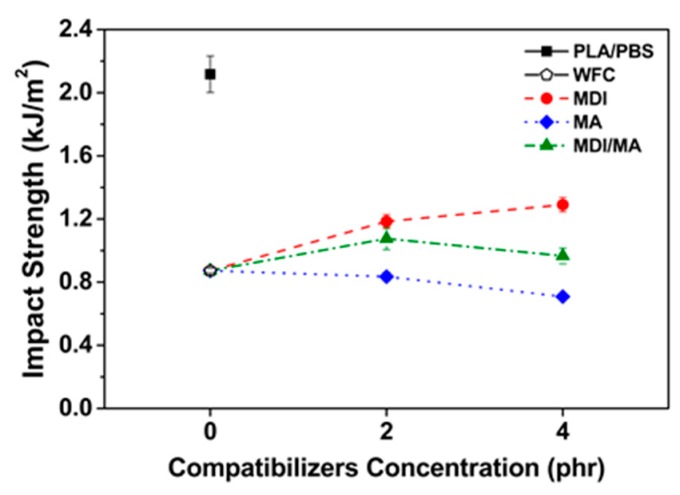
Impact strength of various PLA/PBS/WF biocomposites.

Impact strengths of PLA/PBS/WF biocomposites are shown in Figure 7. The impact strength decreased with the incorporation of WF, likely because of poor interfacial adhesion between hydrophobic polymer matrix and hydrophilic WF, resulting in interrupted the transfer of stress. Also, the WF present in the polymer matrix is related to high stiffness that limits the mobility of the molecular chains. In addition, as the content of PBS with ductility decreases and the content of brittle WF increases, the ability to absorb external impact decreases. The impact strength improved with the incorporation of MDI. This is because the interfacial adhesion was improved by urethane linkage produced by the chemical reaction between isocyanate group of MDI and hydroxyl groups of PLA, PBS and WF [11]. It is also suggested that the elongation at break of tensile properties increased by the incorporation of MDI led to the enhanced ductility of PLA/PBS/WF biocomposites. In contrast, the impact strength decreased with the incorporation of MA. It can be expected that the impact strength will also be increased by improved interfacial adhesion between PLA, PBS, and WF obtained from FT-IR and SEM results. However, the smooth fracture surface by SEM image (Figure 4d) exhibits that the brittleness of PLA/PBS/WF biocomposites is increased. In addition, the elongation at break of tensile properties decreased by the incorporation of MA shows reduced ductility of PLA/PBS/WF biocomposites [18]. From the above results, it can possibly be said that PLA/PBS/WF biocomoposites with MDI/MA have the most balanced mechanical properties compared to PLA/PBS/WF biocomposites with MDI or MA. In addition, the incorporation of excessive coupling agents (4 phr) did not improve the overall mechanical properties of PLA/PBS/WF biocomposites. This suggests that the incorporations of small amounts (2 phr) of coupling agents have a better effect.

### 3.3. Thermal Properties

**Figure 8:**
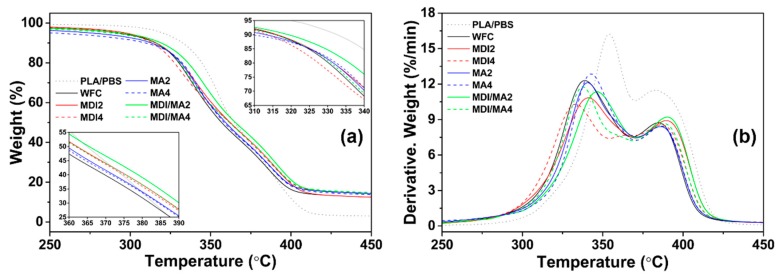
TGA properties of various PLA/PBS/WF biocomposites: (**a**) TG curves and (**b**) DTG curves.

TG and derivative TG (DTG) curves of PLA/PBS/WF biocomposites are presented in Figure 8. The total TGA results for biocomposites are summarized in Table 2. *T*_95_ and *T*_50_ refer to the temperatures at which the remaining composite masses are 95% and 50%, respectively. The first- and second-stage peak maximum temperatures refer to *T*_Max_^1^ by the thermal decomposition of PLA and WF and *T*_Max_^2^ by the thermal decomposition of PBS, respectively [28]. The thermal stability decreased with the incorporation of WF into PLA/PBS matrix. This is because the thermal decomposition of wood (particularly cellulose and hemicellulose) occurs in the temperature range of 200–350 °C; moreover, heat is easily penetrated by poor interfacial adhesion between polymer matrix and WF [29,30]. TG curves of MDI2 shifted to the right compared with WF, and the values of *T*_95_, *T*_50_, *T*_Max_^1^, and *T*_Max_^2^ also increased. This is because the improved interfacial adhesion between polymer matrix and WF with MDI contributed to improved thermal stability [31]. However, MDI4 with an increased amount of MDI exhibited lower thermal stability than MDI2. A maximum saturation concentration value for the surfactant action by the isocyanates of MDI exists; consequently, it means that the incorporation of additional MDI is ineffective for thermal stability [11,25]. The TG curves of MA2 shifted gradually to the right compared with the WF. The values of *T*_50_, *T*_Max_^1^, and *T*_Max_^2^ also increased, while the initial thermal decomposition temperature, *T*_95_, decreased. This indicates that the PLA/PBS/WF biocomposites reacted with maleic anhydride had a lower initial thermal decomposition temperature due to unreacted molecular structures. However, MA caused a gradually cross-linking reaction between the PLA, PBS, and WF by increasing interactions, thereby, enhancing thermal stability. MA4 with an increased amount of MA resulted in a similar (decreased) thermal stability to that of MA2. This is resulted from the increased quantities of MA in the reactive extrusion, causing degradation of the polymer molecule [18]. Also, the molecular weight of polymer is likely to be decreased, because the chain scission by DCP may occur when the MA is incorporated in excess [32]. PLA/PBS/WF biocomposites with MDI/MA exhibited better thermal stability than WFC. Particularly, MDI/MA2 with small amounts of 1 phr MDI and 1 phr MA revealed the most improved thermal stability among the various PLA/PBS/WF biocomposites. It is suggested that the improvement (due to the incorporation of a small amount of MDI and MA coupling agents) is more prominent, and that the synergistic effect is caused by the hybrid incorporation of MDI and MA.

**Figure 9:**
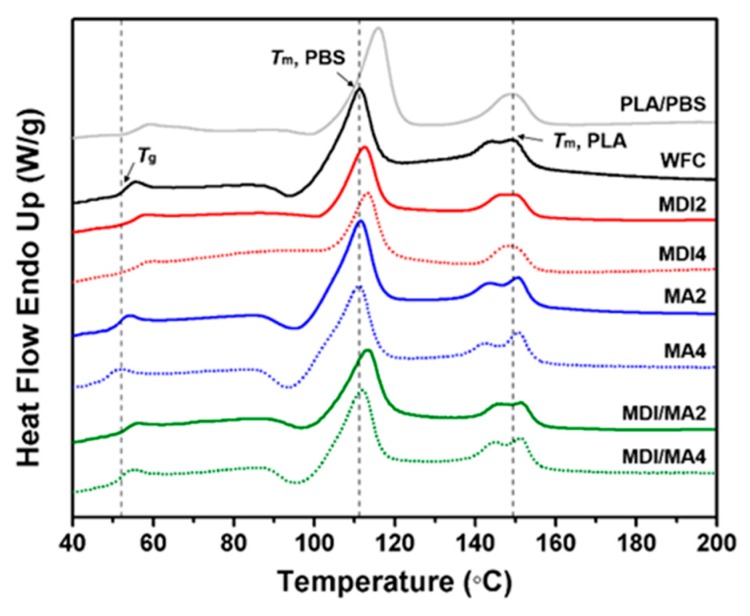
DSC heating run curves of various PLA/PBS/WF biocomposites.

DSC heating run curves of PLA/PBS/WF biocomposites are presented in Figure 9, and the total DSC characteristics for biocomposites are summarized in Table 3. According to the heating run curve of PLA/PBS, *T*_m_ of PLA is 149.20 °C and *T*_m_ of PBS is 115.93 °C. These results are similar to the *T*_m_ values of PLA and PBS reported by Hassan et al. [28]. These two melting endothermic peaks indicate immiscibility between PLA and PBS. *T*_g_ of PLA component in the PLA/PBS/WF biocomposites was lower than that of conventional PLA [33]. It is considered that the PLA/PBS blend had a lower *T*_g_ value overall because PBS with low *T*_g_ accounts for 50% of the total content. Additionally, the *T*_g_ of the PLA/PBS/WF biocomposites was decreased due to the incorporation of WF. This was due to immiscibility as a result of the poor interfacial adhesion between polymer matrix and WF. The *T*_g_ of the PLA/PBS/WF biocomposites increased due to the incorporation of MDI. It is considered that the formation of cross-linked structures by the incorporation of MDI increased the molecular weight, which resulted in high viscosity and consequently, increased the *T*_g_ of biocomposites [34]. Unlike the incorporation of MDI, the *T*_g_ values of PLA/PBS/WF biocomposites were reduced to lower temperatures by the incorporation of MA compared to those without coupling agents. This is probably ascribed to the increased mobility between the molecular chains of polymer and WF by the reaction with MA, resulting in decreased *T*_g_ values due to an increased flow rate. Also, MA may cause the decomposition of PLA and PBS molecules by reactive extrusion as the mentioned above, leading to a decrease in the *T*_g_ values [32,35].

Δ*H*_m_ is an important parameter since its magnitude is directly proportional to the overall level of *X_c_* possessed by polymer. The *X_c_* of PLA and PBS was decreased by the incorporation of WF. The presence of stiff WF particles can increase the viscosity of polymer matrix and limit the crystallization by interfering with molecular chain movement [20]. The *X_c_* of PLA/PBS/WF biocomposites was decreased by the incorporation of MDI. This means that the strong interaction between polymer matrix and WF limits the orientation of polymer chains, resulting in reduced nucleation phenomena. This is related to the PLA melting endothermic peak of the heating run curves appearing as one peak by the incorporation of MDI [11,31]. Therefore, it is suggested that nucleation can be considerably reduced by a strong reaction. The *X_c_* of PLA/PBS/WF biocomposites was increased by the incorporation of MA. This caused the polymers grafted by the incorporation of MA to act as heterogeneous nucleating agents, leading to the creation of nucleation sites for the crystallization process. This is related to the PLA melting endothermic peaks of the heating run curves appearing as two peaks by the incorporation of MA. Furthermore, as the MA content was increased, two distinct peaks appeared. The existence of two melting endothermic peaks was due to the two different types of crystalline lamellae [36]. From these phenomena, the effect of MA supports that the PLA/PBS/WF biocomposites changed from an amorphous to a crystalline material, which resulted in increased *X_c_* [36].

DSC analysis of PLA/PBS/WF biocomposites by the incorporation of MDI or MA revealed differences in thermal behavior. From these different characteristics, the thermal behavior of MDI/MA indicated intermediate results to those presented by the incorporation of MDI or MA.

### 3.4. Viscoelastic Properties

**Figure 10:**
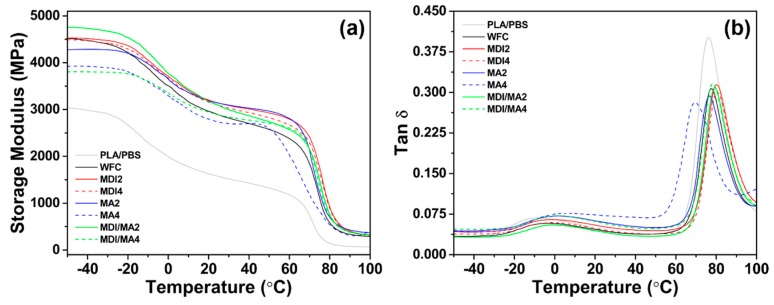
DMA properties of various PLA/PBS/WF biocomposites: (**a**) Storage modulus curves and (**b**) tan δ curves.

Storage modulus and tan δ curves obtained from the DMA data of various PLA/PBS/WF biocomposites are presented in Figure 10. The storage moduli increased significantly with the incorporation of WF. This is probably due to the reinforcing effect imparted to the polymer matrix by the incorporation of WF, which allows the transfer of stress from the polymer matrix to the WF particle [37]. The increase in the storage moduli of thermoplastic polymer-based composites can be attributed to a number of factors, including changes in the crystal–amorphous interface, concentration of filler, particle shape of filler, and the specific area of filler [38]. Therefore, the interfacial miscibility between polymer matrix and filler is a very important factor. As shown in Figure 10a, the storage modulus value for MDI2 was similar to that of WFC at an initial temperature of −50 °C. However, as the temperature increased, the moduli values were higher than those for WFC in an increased temperature range. This supports that the interfacial adhesion improved by the incorporation of MDI maintained a high storage modulus. MDI4 with an increased MDI content exhibited lower storage moduli values than MDI2, which is similar to the mechanical properties results mentioned previously. Therefore, the increase in MDI content did not produce an improvement effect with the coupling agent [11]. Unlike the results for MDI incorporation, the storage modulus of MA2 was lower than WFC in the temperature range of −50 to −20 °C, but was higher than WFC as the temperature increased. MA4 with an increased MA content exhibited significantly lower storage moduli values than WFC in most temperature ranges. These results suggest that although the incorporation of MA improves the interaction between polymer matrix and WF, it may cause plasticization effects and could be related to the decomposition of the polymer matrix [18,39]. The storage modulus value of MDI/MA2 at −50 °C was the highest; the improved storage modulus was better maintained than WFC, even at increasing temperatures. However, MDI/MA4 with an increased coupling agent content showed a reduced storage modulus value.

Tangent delta maximum (tan δ_max_) can also provide information on the *T*_g_ of materials. The tan δ values of the PLA/PBS/WF biocomposites having WF were decreased compared with pure polymer PLA/PBS. This seems to be associated with the flowability of PLA and PBS molecular chains, which are limited by the reinforcing effect of WF, thereby increasing elasticity. At the temperature of the tan δ_max_ peak representing *T*_g_, PLA/PBS/WF biocomposites with MDI showed higher values compared to WFC, but PLA/PBS/WF biocomposites with MA represented lower values compared to the WFC. This was found to be similar to the *T*_g_ results obtained by DSC analysis.

## 4. Conclusions

PLA/PBS/WF biocomposites were processed by reactive extrusion and were successfully compatibilized by the incorporation of MDI, MA, and MDI/MA. Various properties (FT-IR, SEM, mechanical tests, TGA, DSC, and DMA) were analyzed to evaluate the effects of the coupling agents on the biocomposites. Analyses using FT-IR and SEM demonstrated that MDI and MA could achieve a compatible structure between PLA, PBS, and WF. In overall mechanical properties, PLA/PBS/WF biocomposites with MDI represented the most improved features in tensile, flexural, and impact strengths. On the other hand, the highest tensile and flexural moduli values were observed in the PLA/PBS/WF biocomposites with MA. The most balanced mechanical properties between the strength and the moduli occurred in the PLA/PBS/WF biocomposites with both MDI and MA. According to TGA, MDI2 biocomposite with MDI and MA exhibited the most improved thermal stability. In DSC analyses, the incorporation of MDI increased *T*_g_ values and the incorporation of MA increased *X_c_* values. The storage moduli obtained by DMA revealed a more positive effect with the incorporation of MDI than with MA. From the overall results, differences in properties were evident with the incorporation of MDI and MA. The excessive incorporation of coupling agents led to a deterioration in properties. In conclusion, PLA / PBS / WF biocomposites mixed with small amounts of both MDI and MA demonstrated the most balanced properties, and MDI and MA played an important role as coupling agents between PLA, PBS and WF.

## Figures and Tables

**Table 1:** Formulations of various PLA/PBS/WF biocomposites.

Specimen	PLA	PBS	WF	MDI	MA	DCP
	(wt.%)			(phr ^1^)		
PLA/PBS	50	50	−	−	−	−
WFC	35	35	30	−	−	−
MDI2	35	35	30	2	−	−
MDI4	35	35	30	4	−	−
MA2	35	35	30	−	2	0.2
MA4	35	35	30	−	4	0.4
MDI/MA2	35	35	30	1	1	0.1
MDI/MA4	35	35	30	2	2	0.2

^1^ phr: as an abbreviation for parts per hundred resin, this paper represents one part of additives per one hundred parts of PLA/PBS/WF biocomposites.

**Table 2:** TGA results of various PLA/PBS/WF biocomposites with coupling agents.

Specimen	TGA Data			
	*T*_95_ (°C)	*T*_50_ (°C)	*T*_Max_^1^ (°C)	*T*_Max_^2^ (°C)
PLA/PBS	320.0	364.8	354.1	382.5
WFC	292.3	356.9	339.3	385.5
MDI2	296.6	361.7	341.0	388.8
MDI4	294.6	361.8	334.0	386.3
MA2	275.6	359.3	340.4	386.8
MA4	252.6	358.4	343.7	387.1
MDI/MA2	293.3	365.3	347.5	391.5
MDI/MA4	288.4	361.7	338.1	388.3

**Table 3:** DSC characteristics of various PLA/PBS/WF biocomposites with coupling agents.

Specimen	PLA component				PBS component		
	*T*_g_ (°C)	*T*_m_ (°C)	Δ*H*_m_ (°C)	*X_c_* (%)	*T*_m_ (°C)	Δ*H*_m_ (°C)	*X_c_* (%)
PLA/PBS	53.6	149.2	11.9	12.8	115.9	33.1	30.0
WFC	51.1	149.3	10.4	11.2	111.2	28.5	25.9
MDI2	52.6	150.0	7.8	8.4	112.5	19.9	18.1
MDI4	53.1	149.0	6.4	6.9	113.4	16.8	15.2
MA2	49.7	150.7	11.4	12.2	111.4	30.9	28.1
MA4	47.7	150.8	11.6	12.5	111.0	33.5	30.3
MDI/MA2	51.7	151.8	8.2	8.8	112.8	26.7	24.2
MDI/MA4	50.2	151.3	10.9	11.7	111.8	26.4	23.9
